# Accuracy of vestibular schwannoma segmentation using deep learning models - a systematic review & meta-analysis

**DOI:** 10.1007/s00234-024-03449-1

**Published:** 2024-08-24

**Authors:** Paweł Łajczak, Jakub Matyja, Kamil Jóźwik, Zbigniew Nawrat

**Affiliations:** 1 Department of Biophysics, Faculty of Medical Sciences in Zabrze, Medical University of Silesia in Katowice, Jordana 18, Mekelweg 5, Zabrze, 40-043, Poland; 2 TU Delft, Mekelweg 5,, Delft 2628 CD,, Netherlands; 3 Foundation of Cardiac Surgery Development, Zabrze, 41-808 Poland

**Keywords:** Vestibular schwannoma, Acoustic schwannoma, Deep learning, Tumor segmentation, MRI

## Abstract

**Abstract:**

Vestibular Schwannoma (VS) is a rare tumor with varied incidence rates, predominantly affecting the 60–69 age group. In the era of artificial intelligence (AI), deep learning (DL) algorithms show promise in automating diagnosis. However, a knowledge gap exists in the automated segmentation of VS using DL. To address this gap, this meta-analysis aims to provide insights into the current state of DL algorithms applied to MR images of VS.

**Methodology:**

Following 2020 PRISMA guidelines, a search across four databases was conducted. Inclusion criteria focused on articles using DL for VS MR image segmentation. The primary metric was the Dice score, supplemented by relative volume error (RVE) and average symmetric surface distance (ASSD).

**Results:**

The search process identified 752 articles, leading to 11 studies for meta-analysis. A QUADAS- 2 analysis revealed varying biases. The overall Dice score for 56 models was 0.89 (CI: 0.88–0.90), with high heterogeneity (I2 = 95.9%). Subgroup analyses based on DL architecture, MRI inputs, and testing set sizes revealed performance variations. 2.5D DL networks demonstrated comparable efficacy to 3D networks. Imaging input analyses highlighted the superiority of contrast-enhanced T1-weighted imaging and mixed MRI inputs.

**Discussion:**

This study fills a gap in systematic review in the automated segmentation of VS using DL techniques. Despite promising results, limitations include publication bias and high heterogeneity. Future research should focus on standardized designs, larger testing sets, and addressing biases for more reliable results. DL have promising efficacy in VS diagnosis, however further validation and standardization is needed.

**Conclusion:**

In conclusion, this meta-analysis provides comprehensive review into the current landscape of automated VS segmentation using DL. The high Dice score indicates promising agreement in segmentation, yet challenges like bias and heterogeneity must be addressed in the future research.

## Introduction

Vestibular Schwannoma (VS), or Acoustic Schwannoma, is a rare tumor with an incidence rate ranging from 0.3 to 6.1 cases per 100,000 individuals, varying between age groups and ethnicities [1–5]. Mainly affecting individuals in the 60–69 age group, VS causes symptoms such as gradual hearing loss, dizziness, and tinnitus. Symptoms escalate as the tumor increases in size, leading to compression of the cranial nerves, the brainstem, and the cerebellum. This compression results in symptoms such as vomiting, headaches, nausea, or mental confusion [6–8].

Diagnosis of VS is crucial for determining optimal interventions, which include surgery, radiotherapy, or gene therapy. Currently MR scans are considered the gold-standard, utilizing T1 weighted (T1W), T2W weighted (T2W), and contrast-enhanced T1 weighted (ceT1W) image sequences for localization and tumor segmentation assessment [9, 10].

However, as the VS follow-ups span years, manual delineation and segmentation of VS becomes an ineffective, labor-intensive process. Automated segmentation could potentially save a lot of time for physicians, improving the effectiveness of the workplace. Additionally, manual segmentation of the neoplasm is relatively subjective process, which can lead to variations in the results. Currently, challenges associated with limitations of manual segmentation include:

- Clinical decision-making: Accurate volumetric measurements are crucial for monitoring the growth of VS to plan next treatment steps. The time-consuming manual option limits the potential of rapid decisions.
- Superiority of volumetric analysis: 3D volume analysis allows for more comprehensive and accurate analysis than regular linear measurements of the VS. However, due to the time required for analysis, this option remains less attractive.
- Safety of protocols: While contrast scans are still commonly used, implementation of high-resolution scans without contrast for automated segmentation could benefit patients by minimizing the risk of gadolinium accumulation in brain tissues. Additionally, cystic parts are hyperintense on these scans, allowing for better decision making.
- Costs: Automated tools could reduce potential expenses by decreasing the total scanning length and the number of required sequences.

In the era of artificial intelligence (AI), convolutional neural networks (CNNs), a subset of deep learning (DL), demonstrated potential for the automated diagnosis of pathologies. CNNs, which are able to mimic human neurons, have demonstrated promising results in medical applications, including radiology, where DL networks have been applied for detection, tumor volume measurements, and neoplasm segmentation [11–15].

Despite these advancements, currently no work synthesizes information regarding the automated segmentation of VS using DL techniques. Hence, in this study we aim to conduct a systematic review and meta-analysis, describing the current state of algorithms applied to MR image segmentation of this rare neoplasm. By performing such review, we not only aim to fulfill existing gap in synthesis, but also provide insights, limitations, and guidance that may influence the future works related to the topic of automated segmentation of VS.

## Methodology

This study adhered to the 2020 PRISMA guidelines for conducting a systematic review and meta-analysis [16]. The identification of eligible articles involved a comprehensive search across four databases: PubMed, Web of Science, Scopus, and Embase. Our search strategy incorporated MeSH terms and encompassed a range of keywords, including Artificial Intelligence, Deep Learning, Machine Learning, Automation, Convolutional Neural Networks, and specific terms related to vestibular schwannoma, acoustic schwannoma, and perineural fibroblastoma. The complete search strategy is provided in Appendix 1. In addition, a manual review of references was conducted to identify any studies not captured in the initial search. Two authors independently screened articles for inclusion and exclusion based on the outlined criteria, with conflicts resolved through mutual discussion with other authors. The screening process was facilitated by ZOTERO software, employed by both authors.

### Inclusion and exclusion criteria

We established inclusion criteria for our study, including: (I) no restriction on publication date, encompassing articles from all years; (II) inclusion of only original articles in English; (III) exclusion of reviews, abstract-only works, preprints, and other non-original articles; (IV) focus on deep learning MR image segmentation; (V) exclusion of other artificial intelligence techniques such as conventional machine learning (ML); (VI) inclusion of studies with specified validation methods for the meta-analysis, aiming to mitigate overestimation; (VII) inclusion of studies with extractable data for meta-analysis (standard deviation or error), with the Dice score as the primary performance metric, supplemented by data on relative volume error (RVE) and average symmetric surface distance (ASSD).

### Dice score, RVE, ASSD, and other extracted data

The Sørensen-Dice score, or Dice score, quantitatively measures the similarity between two sets, serving as an evaluation metric for segmentation algorithm performance. RVE provides a percentage measure of the deviation between segmented and true volumes, while ASSD gauges the alignment of segmented surfaces with ground truth surfaces (in our study in mm). Additional extracted data from each study included information on country of origin, software and hardware specifications, DL algorithm characteristics, validation types, testing set sizes, MRI inputs, patient demographics, tumor volume, MR imaging specifications, labelling, cystic analysis, training parameters, and dataset source. Independent extraction of data was followed by conflict resolution through discussion with other authors.

### Statistical analysis

Each study underwent individual assessment using the Quality Assessment of Diagnostic Accuracy Studies (QUADAS-2) tool to evaluate the potential risk of bias [17]. Robvis software facilitated the visualization of outcomes [18]. Stata v18 software was employed for statistical analysis, utilizing a random-effects REML model for forest plot construction. Distinct techniques, testing sets, DL algorithms, and MRI inputs were treated as independent results. Subgroup analyses were conducted based on 3-dimensional and 2.5-dimensional architecture algorithms, ceT1W, T2W, T1W and mixed MRI inputs, as well as different testing set sizes (< 50, 50–100, and > 100 patients). Heterogeneity was quantified using the percentage I^2^ index. Significance threshold was set at *p* < 0.05. The analysis employed 95% confidence intervals (CI), and bias was assessed through funnel plot analysis.

## Results

**Fig. 1:**
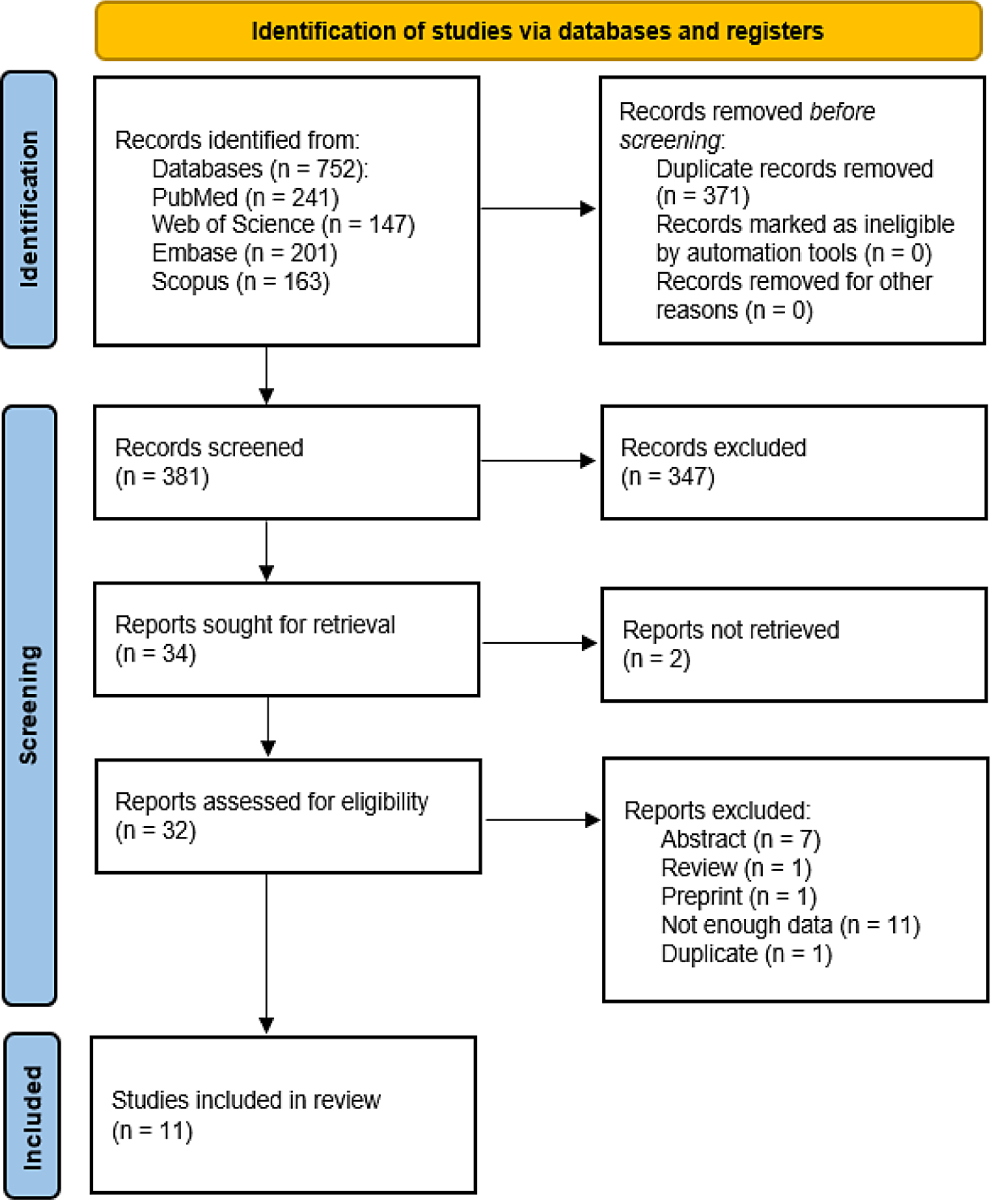
PRISMA flow diagram. Made with PRISMA template [16]

The detailed process of our literature search is outlined in Fig. 1 [16]. We identified a total of 752 articles across four databases: 241 from PubMed, 147 from Web of Science, 201 from Embase, and 163 from Scopus. Using ZOTERO, we initially identified 371 articles to remove duplicates. During screening, two authors independently reviewed 381 records, resolving disagreements through collaboration.

Following screening, we excluded 347 works unrelated to the meta-analysis topic. In the subsequent full-text screening, 34 works were included. Unfortunately, we were unable to retrieve 2 records. For the meta-analysis, we incorporated 11 out of 34 potentially eligible works. Eleven lacked extractable data for statistical analysis, one was a preprint, one had duplicate results, another was a review, and seven were abstract-only articles. Attempts to contact authors via mail correspondence yielded no replies.

### Publication bias

**Fig. 2:**
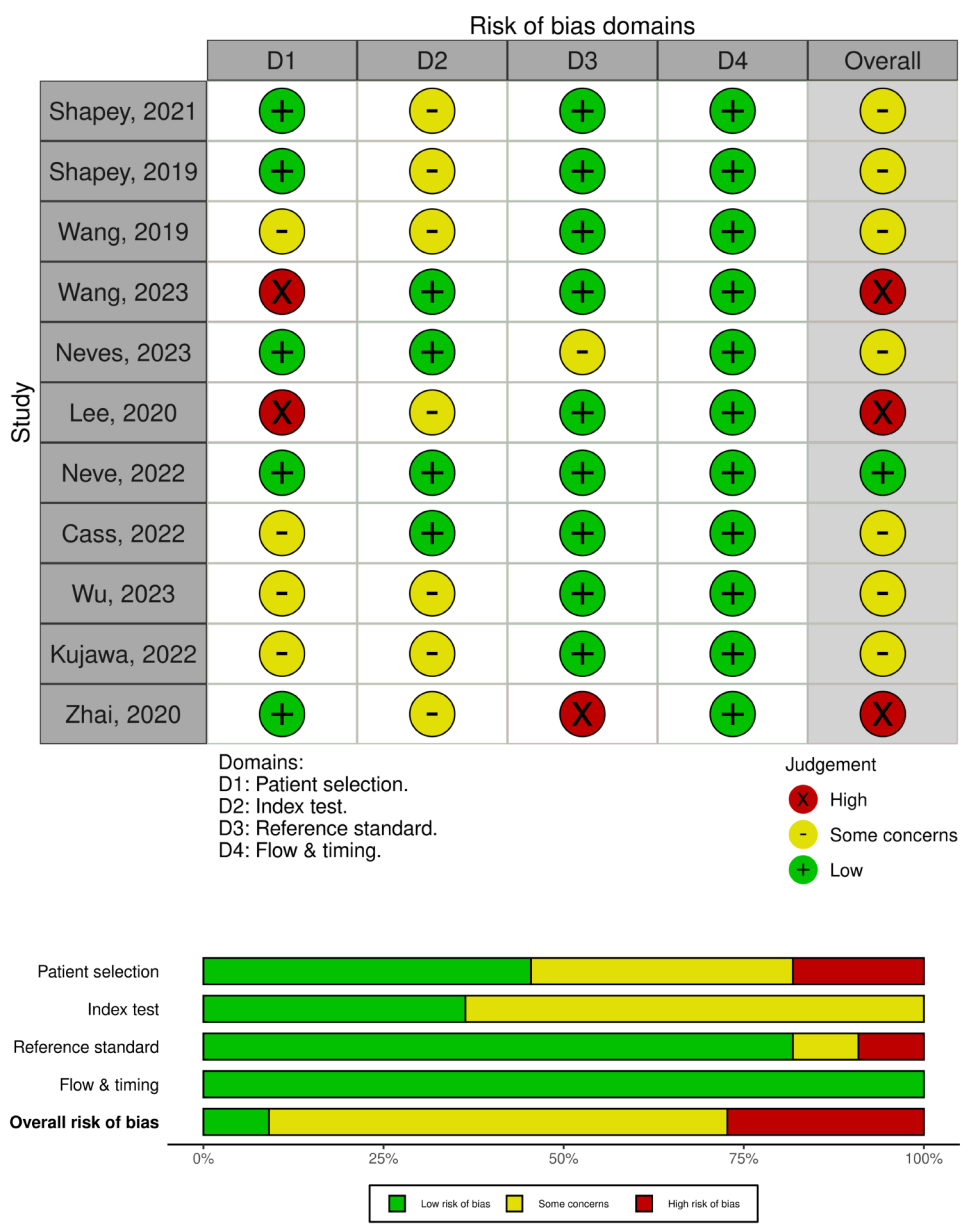
QUADAS-2 chart with assessment of bias of included studies. Made with robvis [18]

We performed a QUADAS-2 analysis using the robvis tool, individually assessing each of eleven studies [19–29]. For each study, we evaluated the patient selection process, index test, reference standard, and flow & timing of the study, with a detailed description available in the QUADAS-2 statement [17]. One article exhibited low risk of bias, seven had some concerns, while three had a high risk of bias. Major concerns were identified in the patient selection process and some in the index test, with no significant concerns in the reference standard and flow and timing section. We have identified lack (or no reports) of clear inclusion or exclusion criteria, unclear process of randomization of the datasets (patients), lack of information of blinding the index test, and two studies had concerns in the labelling process. The QUADAS-2 charts with bias assessment results are presented in Fig. 2.

### Included studies

From the 11 studies included in this meta-analysis, 4 were from the UK, 3 from the USA, 1 from Taiwan, and 1 each from the Netherlands and China, with a collaborative effort between China and the UK. The publication dates ranged from 2019 to 2023. Pytorch and Tensorflow were the most commonly used software, along with NVIDIA GPU cards and Intel CPUs. However, not all studies reported details such as software versions or GPU types. All works were based on CNN platforms, with U-Net being the most popular option, featuring various modifications of modules and dimension processing. The majority of works (6) employed split validation for performance assessment and further tuning. Studies varied in MR imaging techniques, with contrast-enhanced T1-weighted imaging being the most popular, appearing in 9 studies. Detailed information about each study is available in Table 1. We have additionally extracted supplementary data from each study, related to demographics, dose radiation, tumor volume, and MRI specification which is available in the Table 2. Table 3 provides additional information about labelling, training process, and datasets [30, 31].

**Table 1:** Overview of with vestibular schwannoma DL segmentation, included in quantitative review

Study	Software/Hardware	Type of DL	Validation and test size (patients)	MRI	Models (if applicable)
Shapey et al. [19] UK, 2021	PyTorch – MONAI, Insight Toolkit	2.5D U-Net	Split, *n* = 46	ceT1W, T2W	A - ceT1W
					B - T2W
Shapey et al. [20] UK, 2019	Tensorflow. NiftyNet8, Ubuntu desktop − 32 GB RAM, NVIDIA GTX 1080 Ti GPU.	2.5D U-Net, Supervised Attention module (SpvA), hardness-weighted Dice loss (HDL) function	Split, *n* = 46	ceT1W, T2W	A - ceT1W
					B - ceT1W SpvA
					C – ceT1W SpvA + HDL
					D – Ensemble ceT1W
					E – T2W
					F - T2W SpvA
					G – T2W SpvA + HDL
					H – Ensemble T2W
					I - ceT1W + T2W
					J - ceT1W + T2W SpvA
					K – ceT1W + T2W SpvA + HDL
					L – Ensemble ceT1W + T2W
Wang et al. [21] UK, 2019	Tensorflow, NiftyNet, Ubuntu desktop -NVIDIA GTX 1080 Ti GPU	2D, 2.5D & 3D U-Net, SpvA module, Proposed attention module (PA) Dice-Loss, HDL function, Attention gate (AG)	Split, *n* = 47	T2W	
					A − 2.5D U-Net HDL
					B – 2D U-Net
					C – 3D U-Net
					D – 2.5D U-Net + AG
					E – 2.5D U-Net + PA
Wang et al. [22] USA, 2023	PyTorch - MONAI, ResNet block, NVidia Tesla V100 GPU	3D U-Net with SA, deep supervision (DSV) modules	Split from internal and external datasets, *n* = 150	ceT1W	A − 3D U-Net SA
					B − 3D U-Net DSV
					C − 3D U-Net DSV + SA
Neves et al. [23] USA, 2023	Clara SDK, Linux OS, 24 GB GPU RAM	3D ResNet	5-Fold cross, external set *n* = 100	ceT1W	-
Lee et al. [24] Taiwan. 2020	Python 3.6, Tensorflow 1.15, Keras 2.3.1, 2 × 11 GB NVidia RTX 2080Ti GPUs, i7-8700 CPU, 48 GB RAM	3D U-Net single- and 2.5D-like U-Net two-pathway with ReLU	4-Fold cross, *n* = 100	ceT1W, T1, T2W	A - ceT1W, T1, T2W – 1 Pathway (1P) 3D U-Net
					B - ceT1W, T1, T2W – 2 Pathway (2P) 2.5D U-Net
					C - ceT1W − 2P 2.5D U-Net
					D - T1W − 2P 2.5D U-Net
					E - T2W − 2P 2.5D U-Net
					F - T1W + T2W 2.5D U-Net
					G - ceT1W + T2W 2.5D U-Net
Neve et al. [25] Netherlands, 2022	PyTorch 1.7.1, Python 3.8.2, NVIDIA Tesla V100 GPU 16 GB	3D nnU-Net	5-Fold cross, *n* = 23 and *n* = 242 (external dataset)	ceT1W, T2W	A – T2W
					B – ceT1W
					C – ceT1W external dataset
Cass et al. [26] USA, 2022	N/A	3D CNN – CNN and Transformer encoders for Segmentation (CATS)	External, *n* = 30 (No results for internal set)	ceT1W	-
Wu et al. [27] UK, China, 2023	PyTorch, Desktop, Intel i9-10940X CPU, NVIDIA GeForce RTX 2080Ti GPU	2.5D U-Net, loss functions: analysis of prediction and ground truth (fine), probability of error and mis-segmentation (err) coarse segmentation (cor), consistency loss (con)	Split, *n* = 49	ceT1W and T2W	A – real ceT1W
					B - T2W (fine)
					C – ceT1W (fine)
					D – ceT1W (fine) + cor
					E – ceT1W (err) + cor
					F – ceT1W (fine + err) + cor
					G – ceT1W (fine + err + con) + cor
					H – ceT1W + T2W (fine) + cor
					I - ceT1W + T2W (err) + cor
					J - ceT1W + T2W (fine + err) + cor
					K - ceT1W + T2W (fine + err + con) + cor
Kujawa et al. [28] UK, 2022	PyTorch – MONAI 0.50	3D nnU-Net with loss function	5-fold cross, *n* = 62	ceT1W and T2W	A – ceT1W
					B – T2W
					C – ceT1W + T2W
Zhai et al. [29] China, 2023	PyTorch and PyMIC1 on a Ubuntu desktop with an NVIDIA GeForce RTX 3090 GPU	2.5D U-Net, attention U-Net, λ value is a hyperparameter in the Self and Cross Monitoring (SCM) method, controlling the balance between self-training and cross knowledge distillation components	Split, *n* = 46	T2W	A – 2.5D U-Net
					B – 2.5D U-Net λ = 0
					C – 2.5D U-Net λ = 1
					D − 2.5D U-Net λ = 0.5
					E − 2.5D U-Net E λ = 0.5
					F – Attention U-Net
					G – 2.5D U-Net with auxillary 2.5 U-Net
					H – Attention U-Net with auxillary attention U-Net

**Table 2:** Supplementary information from each study

Study	Patients	Male: Female ratio	Age (median [years])	Tumor volume (median [ml])	MR imaging specification	Treatment dose (median)
Shapey, 2021	242	95:147	56 (range 24–84)	Overall: 1.36 (range 0.04–10.78 , IQR 0.63–3.17); Testing: 1.89 (range 0.22–10.78, IQR 0.74–4.05 )	Siemens Avanto 1.5T; ceT1W - MPRAGE sequence (in-plane resolution 0.4 × 0.4, in-plane matrix 512 × 512, slice thickness 1.01.5 (TR = 1900ms, TE = 2.97 ms, TI = 1100 ms)); T2W − 3D CISS sequence (in-plane resolution 0.5 × 0.5, in-plane matrix 384 × 384 or 448 × 448, slice thickness 1.0–1.5 mm (TR = 9.4 ms, TE = 4.23 ms))	13 Gy in 211 cases, 12 Gy in 29 cases, 11 Gy and 15 Gy in one case each
Shapey, 2019	246 (initial 248)	97:151 (2 excluded)	56 (IQR 47–65)	Testing: 1.89 (range 0.22–10.78, IQR 0.74–4.05)	Avanto Siemens 1.5T; ceT1W (in-plane resolution 0.4 × 0.4 mm, in-plane matrix 512 × 512, slice thickness 1.5 mm (TR 1900 msec, TE 2.97 msec, TI [1100 msec)); T2W (in-plane resolution 0.5 × 0.5 mm, in-plane matrix of 384 × 384, slice thickness 1.0–1.5 mm (TR 9.4 msec, TE 4.23 msec))	N/A
Wang, 2019	245	N/A	N/A	N/A	In-plane resolution 0.4 mm × 0.4 mm, in-plane size 512 512, slice thickness 1.5 mm	N/A
Wang, 2023 Institute data	495	239:256	60 (range 13–91)	Overall 0.75 (range 0.03–17.75); Testing 0.69 (range 0.03–11.58)	Siemens 1.5T or 3T; ceT1W – MPRAGE sequence (TR/TE/TI of 4.15/2130/1100 ms [1.5T] and 2.35/2100/900 ms [3T], 3D matrix 256 × 256 × 208, in-plane resolution 0.82 × 0.82 mm, slice thickness 1.0 mm).	12 to 13 Gy
Wang, 2023 Public dataset	242	95:147	56 (range 24–84)	Overall 1.41 (range 0.04–10.78); Testing 1.66 (range 0.07–10.78)	Siemens 1.5T; ceT1W - MPRAGE sequence (in-plane resolution 0.4 × 0.4 mm, slice thickness 1.0–1.5 mm – later in-plane resolution halved and 1.5 mm cross-plane thickness doubled, final resolution of 0.8 × 0.8 × 0.75–1.0 mm)	N/A
Neves, 2023	495 from database, 195 public dataset	N/A	N/A	1.186 (range 0.002–28.461)	N/A	N/A
Lee, 2020	516	N/A	N/A	N/A	GE 1.5 T; ceT1W (Axial 2D Spin Echo (TR = 416 ms, TE = 9 ms, flip angle = 90°), matrix size 512 × 512, voxel size 0.5 × 0.5 × 3 mm); T2W (Axial 2D Spin Echo (TR = 4050 ms, TE = 109 ms, flip angle = 90°), matrix size 512 × 512, voxel size 0.5 × 0.5 × 3 mm)	N/A
Neve, 2022	134	64:70	54 ± 12	N/A	ceT1W (in-plane resolution 0.35 × 0.35, in-plane matrix 400 × 400, TE = 9 ms, TR = 602.1 ms, slice thickness 1 mm); T2W (in-plane resolution 0.29 × 0.29, in-plane matrix 512 × 512, TE = 200 ms, TR = 2400 ms, slice thickness 0.6 mm)	N/A
Cass, 2022	40 from database, 105 from public dataset	N/A	N/A	N/A	N/A	N/A
Wu, 2023	242	N/A	N/A	N/A	N/A	N/A
Kujawa, 2022	308	137:171	57 (IQR 50–67)	N/A	Avanto Siemens Healthineers 1.5T; ceT1W (in-plane resolution 0.4 × 0.4 mm or 0.8 × 0.8 mm, matrix size 256 × 256 or 512 × 512, slice thickness 1.0–1.5 mm, TR 1,900 msec, TE 2.97 msec, TI (inversion time) 1,100 msec); T2W Constructive Interference Steady State (CISS) sequence (in-plane resolution 0.47 × 0.47 mm, matrix size 448 × 448 mm, TR 9.4 = ms, TE = 4.23 msec) or Turbo Spin Echo (TSE) sequence (in-plane resolution 0.55 × 0.55 mm, matrix size 384 × 384 mm, TR = 750 ms, TE = 121 ms, slice thickness 1.0–1.5 mm)	N/A
Zhai, 2023	242	N/A	N/A	N/A	T2W (in-plane resolution 0.5 mm × 0.5 mm, in-plane matrix 384 × 384 or 448 × 448, slice thickness 1.0–1.5 mm − 40–80 slices in a scan)	N/A

**Table 3:** Supplementary information II

Study	Dataset Origin	Training	Labeling; Software	Cystic part analyzed? % of Cystic	Size of VS
Shapey 2021	Public Dataset from Shapey et al. study [30] (Queen Square Radiosurgery Centre, UK)	N/A	GK SRS neurosurgeon, neuroradiologist and physicist; GammaPlan software	N/A	N/A
Shapey 2019	N/A	Adam optimizer; weight decay 10 − 7, batch size 2, iteration number 30 k, learning rate (initial) 10 − 4 and halved every 10 k	Neurosurgeon (x3) and physicist; GammaPlan	N/A	N/A
Wang 2019	N/A	Adam optimizer; weight decay 10 − 7, iteration number 30 k, learning rate (initial) 10 − 4 and halved every 10 k	Neurosurgeon and physicist; GammaPlan	N/A	N/A
Wang 2023	Institution + Public Dataset from Shapey et al. study [30]	Adam optimizer; learning rate (initial) 0.003 and halved every every 100 epochs in the first 200 epochs, and then for every 50 epochs for a total 300 epochs. Rand affine transformation, random image contrast adjustment and Gaussian noise (augmentation).	Neurosurgeons, radiation oncologists and physicists; GammaPlan	Evaluated; N/A	N/A
Neves 2023	Stanford University, USA + Public Dataset from Shapey et al. study [30]	1000 epochs. Rotation up to 20 degrees, elastic deformation, and amplification of 0.9 to 1.1 x (augmentation).	Otolaryngologist	Evaluated; 1% of data set	N/A
Lee 2020	Taipei Veterans General Hospital, Taiwan	Adam optimizer; learning rate 0.001. batch size 4. SGD optimizer; learning rate 0.0001, batch size 4; Total training epoch 40.	Neurosurgeons (x4) and neuroradiologists (x4)	Evaluated; N/A	N/A
Neve 2022	Institution Leiden University Medical Center, Netherlands + Public Dataset from Shapey et al. study [30]	N/A	Physician and technical physician supervised by a senior head-and-neck radiologist; Vitrea software, version 7.14.2.227	Evaluated; 63 (47%)	Intrameatal 28 (21%) Small (0–10 mm) 19 (14%) Medium (11–20 mm) 26 (19%) Moderately large (21–30 mm) 24 (18%) Large (31–40 mm) 24 (18%) Giant (> 40 mm) 13 (10%)
Cass 2022	Datased from crossModa Challenge [31]	N/A	Two experts	N/A	N/A
Wu 2023	Public Dataset from Shapey et al. study [30]	Adam optimizer; batch size 2, learning rate (initial) 1 × 10 − 4 in the first 100 epochs and linearly decayed to 0 in the following 150 epochs.	Neurosurgeon and physicist	N/A	N/A
Kujawa 2022	Public Dataset from Shapey et al. study [30] + Datased from crossModa Challenge [31]	Stochastic gradient descent with Nesterov momentum; 1000 epochs, mini-batch size 2, learning rate (initial) 0.01. Rotations, scaling, Gaussian noise, Gaussian blur, brightness, contrast, simulation of low resolution and gamma correction (augmentation).	Neurosurgeon (x2) and a physicist (x2); GammaPlan	N/A	N/A
Zhai 2023	Public Dataset from Shapey et al. study [30]	Stochastic Gradient Descent; batch size 1, weight decay 3 × 10 − 5, epoch emax 300, learning rate (initial) 10 − 2. Random cropping and flipping along each of the three axes (augmentation).	N/A	N/A	N/A

### Overall performance analysis

**Fig. 3:**
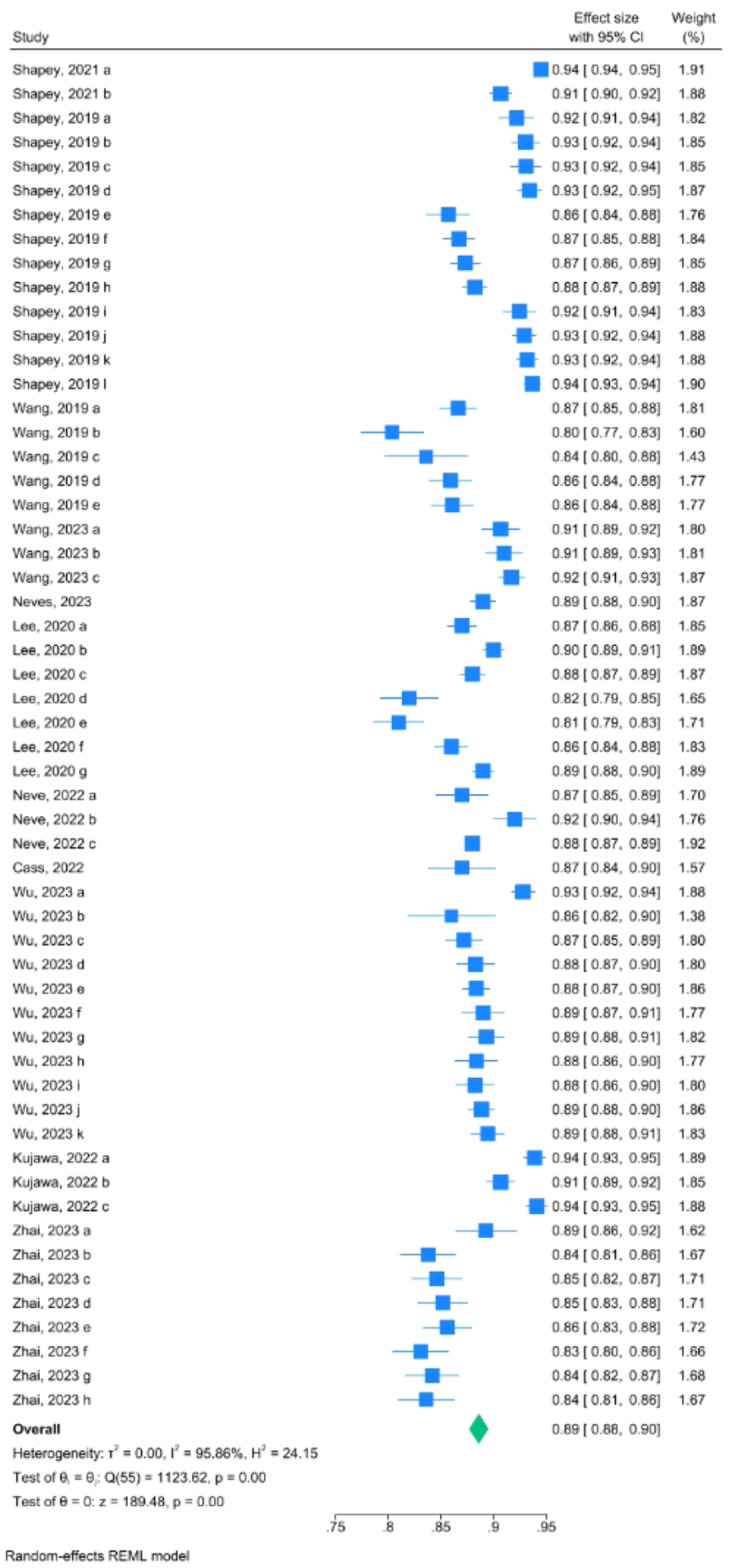
Forest plot with overall mean Dice score pooled from 12 studies

**Fig. 4:**
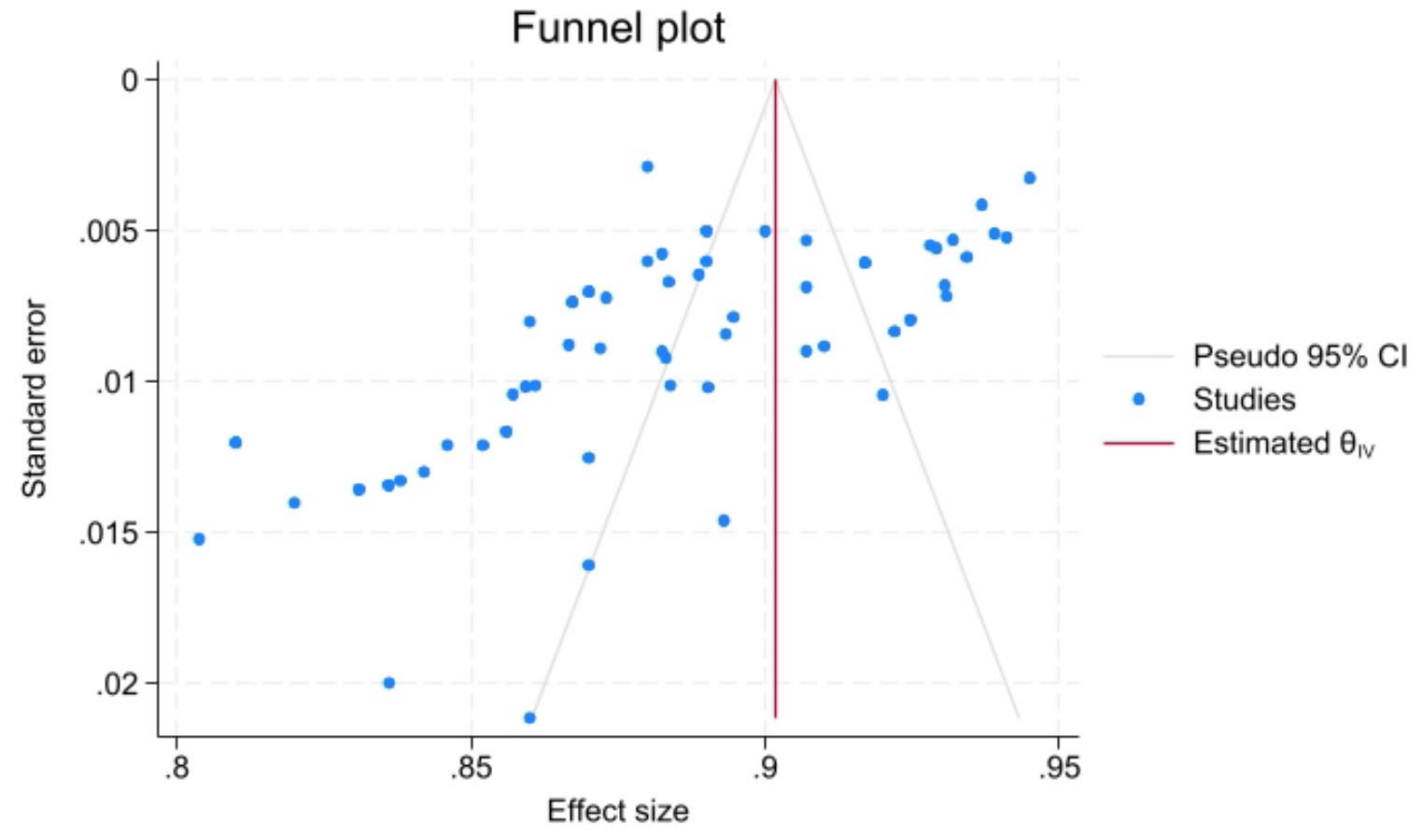
Funnel plot for overall Dice score

Regarding the overall performance of segmentation, fifty-six models from eleven studies reported a mean Dice score for VS MR image segmentation. The pooled mean Dice score was 0.89 (CI: 0.88–0.90, *p* < 0.001), with a heterogeneity of 95.9% (I^2^). Forest plot with overall Dice results is provided in Fig. 3. The lowest reported value was 0.80, while the highest was 0.94. A funnel plot revealed asymmetry (Fig. 4).

The pooled ASSD was 0.46 (CI: 0.42–0.51, *p* < 0.001), with a heterogeneity (I^2^) of 96.1%, from 43 models. The mean relative volume error was 11.7% (CI: 9.7–13.7, *p* < 0.001) from 24 models, with a heterogeneity (I^2^) of 93.8%.

### Architecture subgroup analysis

In the architecture subgroup analysis, we compared different DL architectures, mainly 2.5D networks (40 models), 3D networks (13 models), and other models that did not fit into these categories (3). Among the 2.5D DL networks, the mean Dice score was 0.89 (CI: 0.88–0.90), with a heterogeneity of 94.4% (I^2^). Thirty-two models reported ASSD, and the pooled result was 0.45 (CI: 0.40–0.50, I^2^ = 94.9%). The reported RVE was 10.3% (CI: 8.7–11.9, I^2^ = 86.6%) from 15 models.

For 3D networks, the pooled Dice score was 0.90 (CI: 0.88–0.91), with a heterogeneity of 94.5%. From 8 models, the average ASSD was 0.45 (CI: 0.33–0.56, I^2^ = 95.7%), and the pooled RVE was 13.4% (CI: 8.7–18.0, I^2^ = 95.8%).

For other networks, the mean Dice score was 0.82 (CI: 0.81–0.84), with I^2^ = 26.2%, and ASSD = 0.72 (CI: 0.55–0.88, I^2^ = 76.2%). Only one work pooled RVE, 18.0% (CI: 13.1–22.9).

### MRI input subgroup analysis

In the MRI input subgroup analysis, models were divided into groups based on their imaging input. The mixed group was for DL networks using two or more different MRI inputs. The mean Dice score for 13 models using mixed MR imaging inputs was 0.90 (CI: 0.89–0.92, I^2^ = 94.9%), and the mean ASSD from 8 models was 0.37 (CI: 0.28–0.45, I^2^ = 94.7%). Four models reported RVE, pooling 7.8% (CI: 6.9–8.6, I^2^ = 0.0%).

For 22 T2W models, the mean dice score was 0.86 (CI: 0.85–0.87, I^2^ = 86.3%), 19 models reported ASSD 0.59 (CI: 0.54–0.64, I^2^ = 70.4%). Pooled RVE from 10 models was 14.0% (CI: 12.3–15.6, I^2^ = 57.6%).

Twenty models used ceT1W input – pooled Dice score was 0.91 (CI: 0.90–0.92, I^2^ = 93.5%). From 16 models reporting ASSD, mean results pooled was 0.36 (CI: 0.31–0.41, I^2^ = 94.5%). Ten models reported RVE, yielding 10.9% (CI: 7.1–14.6, I^2^ = 96.7%).

Only 1 model used T1W input and only reported a Dice score of 0.82 (CI: 0.79–0.85).

### Testing set size subgroup analysis

In the final subgroup analysis, we compared models’ performance, grouping them based on their testing set size. DL algorithms were divided into < 50, 50–100, and > 100 patient set sizes. There were 41 models tested on sets smaller than 50. The pooled Dice score was 0.88 (CI: 0.87–0.90, I^2^ = 95.0%). Thirty-eight models reported ASSD, and the pooled result was 0.48 (CI: 0.43–0.53, I^2^ = 95.4%), while the mean RVE from 19 models was 11.0% (CI: 9.4–12.6, I^2^ = 87.1%).

Eleven models were tested on sets of size 50–100 patients. The pooled Dice score was 0.88 (CI: 0.86–0.91, I^2^ = 97.6%). One work reported ASSD and RVE.

**Fig. 5:**
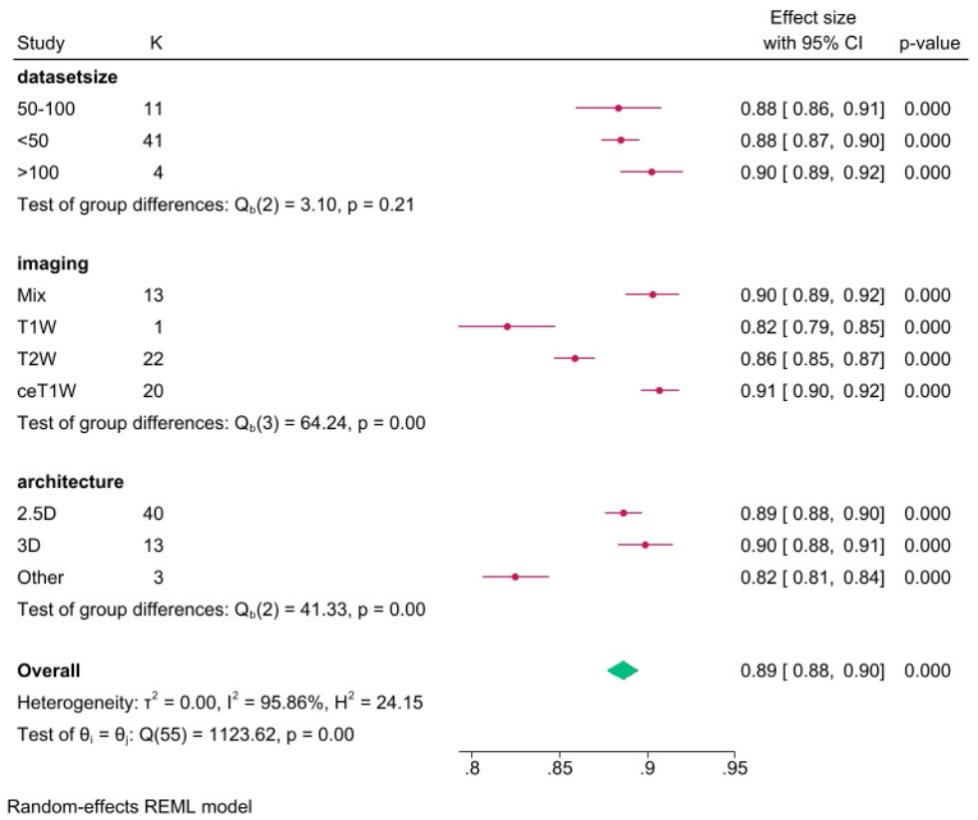
Dice score subgroup analysis results

**Fig. 6:**
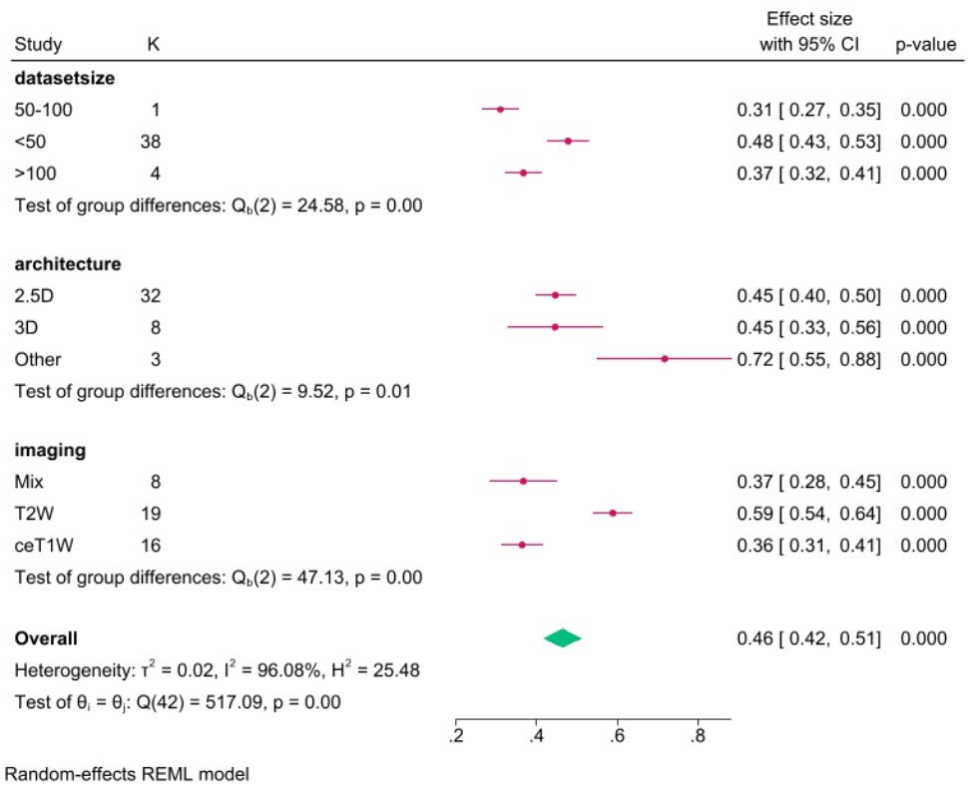
ASSD (mm) subgroup analysis results

**Fig. 7:**
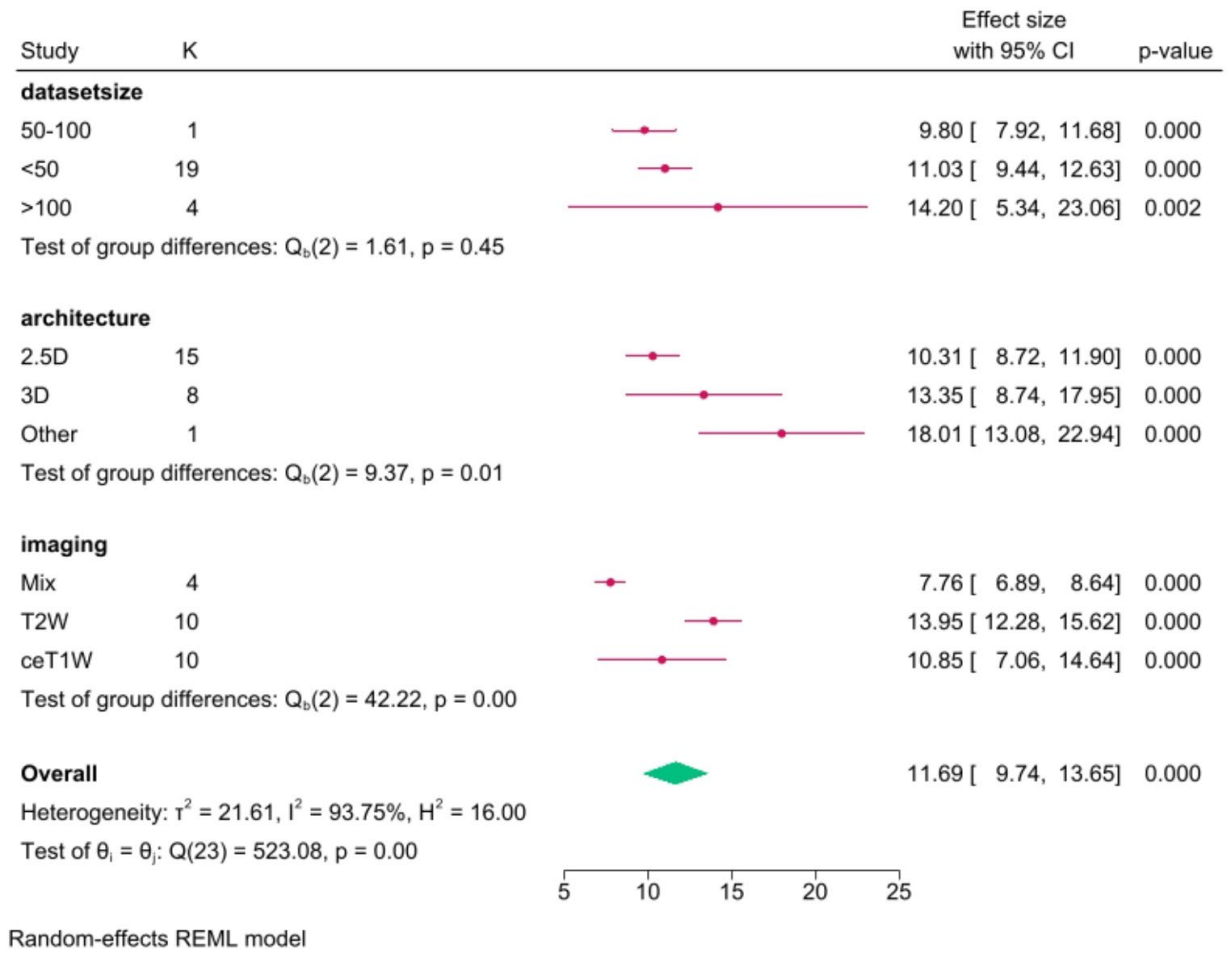
RVE (%) subgroup analysis results

Four models were tested on sets with more than 100 patients. The pooled Dice score was 0.90 (CI: 0.89–0.92, I^2^ = 88.2%). Pooled ASSD was 0.37 (CI: 0.32–0.41, I^2^ = 45.1%), while RVE was 14.2% (CI: 5.3–23.1, I^2^ = 98.3%). A Dice score subgroups analysis summary is available as Fig. 5, for ASSD subgroups as Fig. 6, while for RVE as Fig. 7.

## Discussion

In the rapidly evolving landscape of medical imaging, our systematic review and meta-analysis aims to comprehensively summarize the current state of automated segmentation of Vestibular Schwannoma using Deep Learning techniques. As artificial intelligence, particularly Convolutional Neural Networks, continues to redefine diagnostic paradigms, understanding the application and reliability of these technologies is imperative.

The rarity of VS and its potential severity underscore the importance of accurate and timely diagnosis. Our meta-analysis emphasizes the significance of advanced imaging techniques, primarily MR scans, in evaluating tumor localization and size. Diagnosis of rare tumors might be especially challenging for inexperienced residents of radiology. The implementation of DL not only assists younger specialists but also reduces the time required for the manual segmentation process. Traditional gold standard methods, while effective, are being complemented and, in some instances, challenged by the advent of AI. Reduced workload allows for routine application of volumetric analysis [32]. Manual contouring suffers from inter-operator bias [24]. Automated segmentation offers the potential for standardized and reproducible volume measurements. Traditional linear measurements lack sensitivity, while volumetric measurements are more accurate but much more time-consuming [33, 34]. DL automation allows for efficient and accurate 3D volume calculation, facilitating better clinical decision-making. It is estimated, that automation tools could reduce even 30% time for segmentation [35]. Finally, the ability to perform segmentation using solely T2-weighted MRI addresses concerns about gadolinium contrast agents and potentially reduces costs, to an even 10-fold saving per scan [36, 37].

The integration of CNNs, a subset of DL, holds promise in automating the diagnostic process for VS. The substantial number of MRI scans associated with VS diagnosis and follow-up contributes significantly to healthcare costs. AI-based segmentation offers a novel option to address these conditions by optimizing the workflow of radiologists. Our meta-analysis attests to the increasing interest in leveraging DL for condition detection, tumor volume measurements, and neoplasm segmentation.

Despite strides in AI applications in medical imaging, our study reveals a critical knowledge gap concerning automated segmentation of VS using DL techniques. To the best of our knowledge, this is the very first systematic review and meta-analysis on this topic.

Adherence to the 2020 PRISMA guidelines and the use of the QUADAS-2 tool underscore the methodological rigor employed in our study. Comprehensive searches across major databases, incorporation of specific keywords, and manual reviews of references aimed to encompass relevant literature. Our inclusion criteria, while necessary, led to the exclusion of studies lacking extractable data, highlighting the importance of standardized reporting in future research.

The pooled mean Dice score of 0.89 indicates a commendable level of agreement between segmented and true volumes. However, the observed heterogeneity of 95.9% raises questions about methodological consistency across studies. High heterogeneity could be influenced by the different number of patients used in testing, training and validating models, population (patient) differences, different functions applied in the DL architecture, as well as technical differences in the MR images.

While the Dice score provides a comprehensive measure of segmentation accuracy, the inclusion of Relative Volume Error and Average Symmetric Surface Distance adds granularity to our assessment. The pooled RVE of 11.7% indicates the average deviation between segmented and true volumes. The ASSD of 0.46 mm highlights the alignment of segmented surfaces with ground truth surfaces, showcasing the robustness of DL algorithms in preserving anatomical details.

The subgroup analyses further illuminate the nuanced performance variations. 2.5D DL networks, with a mean Dice score of 0.89, demonstrate comparable efficacy to 3D networks (mean Dice score of 0.90) and outperform other models (mean Dice score of 0.82). Imaging input analyses reveal the superiority of contrast-enhanced T1-weighted imaging and mixed MRI inputs, emphasizing the influence of input modality on segmentation outcomes.

Studies varied in the MRI imaging protocols. Scanner field strength ranged from 1.5T to 3T. For the ceT1 scans, MPRAGE sequence was the most common option. T2W weighted scans utilized 3D CISS, TSE, and 2D Spin Echo sequences. However, notable variations in slice thickness and in-plane resolution (Table 2) could affect the effectiveness of the algorithms in other scenarios.

Despite the promising findings, limitations exist. We were unable to retrieve two records, what arises publication bias. Through QUADAS-2 tool, we have identified bias in different aspects of included studies – these risks in included works should be acknowledged, as well as high heterogeneity of the results. Additionally, articles only in the English language were included. Readers should be aware of these limitations, and future systematic reviews should address these gaps.

Regarding external validation, most of the studies did not perform external analysis of the AI, which raises the risk of overfitting to certain MRI setups. Additionally, external datasets mostly relied on a single database provided by Shapey et al. [30]. This raises question how will DL models perform in the randomized trials or prospective studies with different environments. Studies also lacked software (type and version used) and/or hardware information (GPU type), which could guide future researchers. These aspects are useful for software designers, and should be reported in the future studies.

Another aspect is the analysis of the cystic and solid parts of the VS. As the imaging properties differ, not all studies have deeply analysed them, and often only mentioned difficulties with cystic parts analysis [38]. Finally, other parameters such as size (beside volume), and proportion of the cystic cases should be provided. These aspects are crucial to be resolved in the future trials to provide more comprehensive analysis of VS for better clinical decision-making.

While the high Dice score shows promising results, which could be theoretically implemented in clinical practice, currently due to the limitations, more testing is required for full-scale clinical application. Future research should report fully validated models with more standardized designs, in order to increase homogeneity across the studies. Additionally, we believe that using bigger testing sets, would decrease overestimation of the results, as majority of DL models in this study were assessed on sets with 100 or less patients.

## Conclusions

To summarize, this meta-analysis explores current knowledge of automated segmentation of VS using DL techniques. While Dice, RVE and ASSD scores show promising results for this task, the study is limited by high heterogeneity, caused by variations in MRI techniques, population differences and DL model differences, as well as bias among studies. Future research in this field should address these limitations for more standardized results.

## Data Availability

Authors declare, that all relevant data was presented within the manuscript or figures. On special request, authors are able to send files with data used in this meta-analysis.

## Appendix 1

Search strategy:

1. PUBMED/Scopus/Web of science(AI OR ( artificial AND intelligence ) OR automation OR ( convolutional AND neural AND network ) OR cnn OR ( deep AND learning ) OR ( machine AND learning ) OR ( decision AND tree) OR ( neural AND network ) OR (support AND vector AND machine) OR lasso OR (k-means) OR knn) AND (Vestibular Schwannoma OR acoustic neuroma OR (acoustic AND neuroma) OR (Vestibular AND Schwannoma) OR acoustic neurilemmoma OR neurinoma of the acoustic nerve OR schwannoma of the acoustic nerve OR perineural fibroblastoma).
2. Embase
  (ai OR (artificial AND (‘intelligence’/exp OR intelligence)) OR ‘automation’/exp OR automation OR (convolutional AND neural AND (‘network’/exp OR network)) OR cnn OR (deep AND (‘learning’/exp OR learning)) OR ((‘machine’/exp OR machine) AND (‘learning’/exp OR learning)) OR ((‘decision’/exp OR decision) AND (‘tree’/exp OR tree)) OR (neural AND (‘network’/exp OR network)) OR ((‘support’/exp OR support) AND (‘vector’/exp OR vector) AND (‘machine’/exp OR machine)) OR ‘lasso’/exp OR lasso OR ‘k means’/exp OR ‘k means’ OR knn) AND (‘vestibular schwannoma’/exp OR ‘vestibular schwannoma’ OR ‘acoustic neuroma’/exp OR ‘acoustic neuroma’ OR (acoustic AND (‘neuroma’/exp OR neuroma)) OR (vestibular AND (‘schwannoma’/exp OR schwannoma)) OR ‘acoustic neurilemmoma’ OR (acoustic AND (‘neurilemmoma’/exp OR neurilemmoma)) OR 'neurinoma of the acoustic nerve’ OR ((‘neurinoma’/exp OR neurinoma) AND of AND the AND acoustic AND (‘nerve’/exp OR nerve)) OR 'schwannoma of the acoustic nerve’ OR ((‘schwannoma’/exp OR schwannoma) AND of AND the AND acoustic AND (‘nerve’/exp OR nerve)) OR ‘perineural fibroblastoma’ OR (perineural AND (‘fibroblastoma’/exp OR fibroblastoma)))
