# Correction to: Accuracy of vestibular schwannoma segmentation using deep learning models - a systematic review & meta-analysis

**DOI:** 10.1007/s00234-026-04056-y

**Published:** 2026-06-03

**Authors:** Paweł Łajczak, Jakub Matyja, Kamil Jóźwik, Zbigniew Nawrat

**Affiliations:** 1 Department of Biophysics, Faculty of Medical Sciences in Zabrze, Medical University of Silesia in Katowice, Jordana 18, Mekelweg 5, Zabrze, 40-043 Poland; 2 TU Delft, Mekelweg 5, Delft, 2628 CD Netherlands; 3 Foundation of Cardiac Surgery Development, Zabrze, 41-808 Poland

**Correction to: Neuroradiology (2025) 67:729–742**

**https://doi.org/10.1007/s00234-024-03449-1**

The original version of this article was revised.

In the Declaration section, “Declaration of Generative AI and AI-assisted technologies in the writing process” has been added.

The complete statement should read as follows:

**Declaration of Generative AI and AI-assisted technologies in the writing process** During the preparation of this work, the authors used Gemini (Google Inc.) during writing and grammar checking process. After using this tool, the authors reviewed and edited the content as needed and take full responsibility for the content of the published article. A large language model was not used for data extraction, screening, or statistical analysis. All ideas, analysis, and final content were produced and approved by the authors.

The original article has been corrected.

